# Theory and classification of mass extinction causation

**DOI:** 10.1093/nsr/nwad237

**Published:** 2023-09-08

**Authors:** Thomas J Algeo, Jun Shen

**Affiliations:** State Key Laboratory of Biogeology and Environmental Geology, China University of Geosciences—Wuhan, Wuhan430074, China; State Key Laboratory of Geological Processes and Mineral Resources, China University of Geosciences—Wuhan, Wuhan430074, China; Department of Geosciences, University of Cincinnati, Cincinnati, OH45221, USA; State Key Laboratory of Geological Processes and Mineral Resources, China University of Geosciences—Wuhan, Wuhan430074, China

**Keywords:** biocrisis, ultimate cause, proximate cause, bolide, large igneous province, bioevolutionary event

## Abstract

Theory regarding the causation of mass extinctions is in need of systematization, which is the focus of this contribution. Every mass extinction has both an ultimate cause, i.e. the trigger that leads to various climato-environmental changes, and one or more proximate cause(s), i.e. the specific climato-environmental changes that result in elevated biotic mortality. With regard to ultimate causes, strong cases can be made that bolide (i.e. meteor) impacts, large igneous province (LIP) eruptions and bioevolutionary events have each triggered one or more of the Phanerozoic Big Five mass extinctions, and that tectono-oceanic changes have triggered some second-order extinction events. Apart from bolide impacts, other astronomical triggers (e.g. solar flares, gamma bursts and supernova explosions) remain entirely in the realm of speculation. With regard to proximate mechanisms, most extinctions are related to either carbon-release or carbon-burial processes, the former being associated with climatic warming, ocean acidification, reduced marine productivity and lower carbonate δ^13^C values, and the latter with climatic cooling, increased marine productivity and higher carbonate δ^13^C values. Environmental parameters such as marine redox conditions and terrestrial weathering intensity do not show consistent relationships with carbon-cycle changes.

In this context, mass extinction causation can be usefully classified using a matrix of ultimate and proximate factors. Among the Big Five mass extinctions, the end-Cretaceous biocrisis is an example of a bolide-triggered carbon-release event, the end-Permian and end-Triassic biocrises are examples of LIP-triggered carbon-release events, and the Late Ordovician and Late Devonian biocrises are examples of bioevolution-triggered carbon-burial events. Whereas the bolide-impact and LIP-eruption mechanisms appear to invariably cause carbon release, bioevolutionary triggers can result in variable carbon-cycle changes, e.g. carbon burial during the Late Ordovician and Late Devonian events, carbon release associated with modern anthropogenic climate warming, and little to no carbon-cycle impact due to certain types of ecosystem change (e.g. the advent of the first predators around the end-Ediacaran; the appearance of Paleolithic human hunters in Australasia and the Americas). Broadly speaking, studies of mass extinction causation have suffered from insufficiently critical thinking—an impartial survey of the extant evidence shows that (i) hypotheses of a common ultimate cause (e.g. bolide impacts or LIP eruptions) for all Big Five mass extinctions are suspect given manifest differences in patterns of environmental and biotic change among them; (ii) the Late Ordovician and Late Devonian events were associated with carbon burial and long-term climatic cooling, i.e. changes that are inconsistent with a bolide-impact or LIP-eruption mechanism; and (iii) claims of periodicity in Phanerozoic mass extinctions depended critically on the now-disproven idea that they shared a common extrinsic trigger (i.e. bolide impacts).

## Teaser

This study examines how best to identify and classify the causes of mass extinction events. It recognizes not only the intensely studied bolide impact and large igneous province eruption mechanisms of mass extinction, but also bio-evolutionary and tectono-oceanic mechanisms that have been given inadequate consideration to date.

## HISTORY OF MASS EXTINCTION RESEARCH

### Recognition and naming of mass extinctions

Mass faunal extinctions in Earth history have been the focus of much recent research, stimulated in large measure by a fascination with the biotic impacts of catastrophes and what they might portend for the future. Yet the idea that a species could go extinct is a relatively new one, first proposed by anatomist Georges Cuvier in a 1796 lecture about mastodons [1]. Research on extinctions was limited during the early 19th century owing to prevailing views guided by religious dogma (i.e. that Earth's biota had been created by a supreme being and was therefore immutable) or uniformitarian principles (i.e. species evolved and died out over geologically long intervals, e.g. Charles Lyell and Charles Darwin). In 1841, John Phillips formalized the division of the Phanerozoic into three eras separated by mass extinctions at the ends of the Permian and Cretaceous periods, representing a milestone in extinction research. Over the course of the 19th century, extinction events came to define the boundaries between most first- and second-order subdivisions (i.e. eras and periods) of the Phanerozoic portion of the geologic timescale.

It is now well-established that there have been numerous mass extinctions in the Earth's past, the most thoroughly studied of which are the so-called ‘Big Five’ Phanerozoic events [2], herein termed the Late Ordovician (LOME), Late Devonian (LDME), end-Permian (EPME), end-Triassic (ETME) and end-Cretaceous (ECME) mass extinctions. It should be noted that the LDME is conventionally regarded as two separate extinctions separated by ∼12 Myr, i.e. the end-Frasnian and end-Devonian events (which record similar environmental responses, although identical triggers should not be assumed), although the ∼30-Myr interval from the late Early Devonian to the Devonian-Carboniferous boundary encompasses both as well as ∼8–10 smaller bioevents [3]. For the Phanerozoic as a whole, another ∼15 second-order biocrises might qualify as mass extinctions [4], among which the late Cambrian, Early Carboniferous, end-Guadalupian, Early Toarcian and end-Cenomanian are especially prominent. Note that, for the sake of systematization, several conventions are adopted in this review: (i) extinctions are named for the terminal geologic period or stage rather than for the boundary itself—thus, ‘ECME’ is adopted in place of ‘Cretaceous–Paleocene boundary (KPB)’, ‘end-Frasnian’ in place of ‘Frasnian–Famennian boundary (FFB)’ and so forth, and (ii) extinctions occurring <1 Myr and >1 Myr prior to the boundary of interest are termed ‘end-’ and ‘Late’, respectively [cf. 5]. Finally, most biologists and Earth scientists accept that a ‘Sixth Mass Extinction’ is presently underway, which we will refer to as the ‘Late Quaternary mass extinction’ (LQME).

### Causation of mass extinctions

The earliest ideas concerning mass extinctions imputed biblical causation (e.g. Noachian floods, as propounded by Oxford don William Buckland), a cultural meme that lingers in creationist communities today. Throughout most of the 20th century (i.e. pre-1980), the prevailing scientific paradigms for mass extinctions invoked vaguely defined changes in ocean salinity [6], sea level [7] or plate tectonics [8], and some of these mechanisms have continued to draw adherents even in more recent times (e.g. the sea-level advocacy of [9]). From the modern perspective, however, these mechanisms are highly improbable as agents of biotic annihilation given that such changes have occurred almost continuously throughout Earth history and generally at rates far too slow to serve as the trigger of a biocrisis.

The modern phase of mass extinction research effectively began in 1980 with the seminal paper by Alvarez *et al.* [10] on an extraterrestrial cause for the ECME, which was then known as the ‘Cretaceous–Tertiary boundary (KTB) mass extinction’. Although the concept of mass extinction by bolide (meteor) impact had been proposed earlier [11], the Alvarez *et al.* study was transformative in identifying for the first time the geochemical and petrographic signatures of such an event, validating the concept of ‘neocatastrophism’. These signatures, e.g. iridium anomalies, microspherule layers, quartz shock lamellae and fused breccia, are now standard features by which bolide impacts are recognized in the rock record [12]. Although not diagnostic of a bolide impact, a negative carbon isotope excursion (NCIE) in marine carbonates during the earliest Paleocene was linked to a short-term productivity collapse, demonstrating the scale of ecosystem disruption potentially unleashed by a global catastrophe [13]. The Alvarez *et al.* study also stimulated much later research on the ECME, an overwhelming share of which has served to further substantiate the bolide impact model, a crowning achievement being the discovery of the impact site at Chicxulub, Mexico [14].

**Figure 1.**
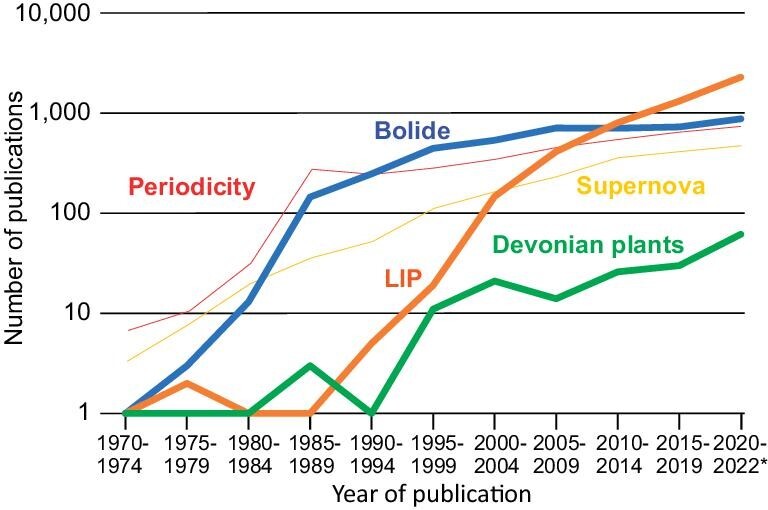
Results of Google Scholar searches (February 2023) for ‘mass extinction’ combined with each of the five terms shown in the figure. Interest in bolides increased sharply after publication of [10], with similar rising trends for LIPs and Devonian plants after key papers proposing those triggers for mass extinctions in the early 1990s. Note: ‘Devonian plants’ was used in preference to ‘Bioevolutionary trigger’, as the latter more general term is not in widespread usage; papers on bioevolutionary mechanisms are thus undercounted in this survey. The large numbers of papers examining mass extinctions in the context of supernovas and periodicity are sourced disproportionately from the astrophysical community (e.g. [15]) and reflect an inadequate understanding of the current state of knowledge in the Earth sciences community regarding mass extinction causation. The numbers of publications for the 2020–2022 interval (*) have been multiplied by 5/3 to match the 5-year periods of the preceding time bins.

Since 1980, research on the causation of mass extinctions has been characterized by a ‘bandwagon effect’, in which a model developed for one specific mass extinction is claimed to apply to many or all biocrises, reducing agency to a single cause. For example, in the decade following publication of the Alvarez *et al.* study, the bolide model dominated research on mass extinctions (Fig. 1), with all of the Big Five mass extinctions as well as some smaller biocrises being attributed to this mechanism ([16] and many other studies). However, extensive hunts for impact-related features at other (i.e. non-ECME) mass extinction horizons yielded few affirmative results, with much of the putatively bolide-related evidence having a limited spatial distribution and/or not coinciding with the extinction horizon proper. For example, research on the LDME yielded various undiagnostic carbon-sulfur isotopic anomalies [17,18], minor Ir anomalies at some stratigraphic remove from the FFB [19,20] and scattered microspherules of uncertain provenance [21,22], as well as identification of two impact craters (Siljan Ring, Sweden; Alamo Crater, Nevada, USA) that were later shown to substantially predate (by ≥5 Myr) the end-Frasnian biocrisis [23,24]. Particularly troublesome is that, in the competition to find evidence of bolide impacts, insufficiently vetted research was published, e.g. the claim of extraterrestrial ^3^He/^4^He ratios at the Permian–Triassic boundary [25], a finding that later proved to be irreproducible [26]. Although limited advocacy of the bolide impact mechanism for non-ECME events persists to the present, e.g. [27], the paucity of evidence for impacts in conjunction with the end-Frasnian and other biocrises eventually became apparent, and the search for mechanisms of mass extinction moved on to new ideas.

The most recently proposed extinction mechanism to be given the ‘bandwagon’ treatment is large igneous province (LIP) eruptions. Interest in LIPs accelerated rapidly with the introduction of this term in the early 1990s [28], and it now represents the most popular hypothesis for mass extinction causation (Fig. 1). LIPs were quickly linked to the EPME and ETME biocrises, i.e. the Siberian Traps [29] and the Central Atlantic Magmatic Province [30], respectively, and much subsequent work has substantiated their role in these biocrises (reviewed in [31]). On the strength of these two well-documented examples, a general theory of LIP eruptions as the trigger for all major Phanerozoic biocrises has been advanced several times [32–34]. Yet despite much subsequent research, evidence for LIP eruptions of sufficient magnitude to trigger a global biocrisis remains sparse for the other Big Five mass extinctions, and the role of LIPs in these events remains speculative and contentious (see below).

**Figure 2.**
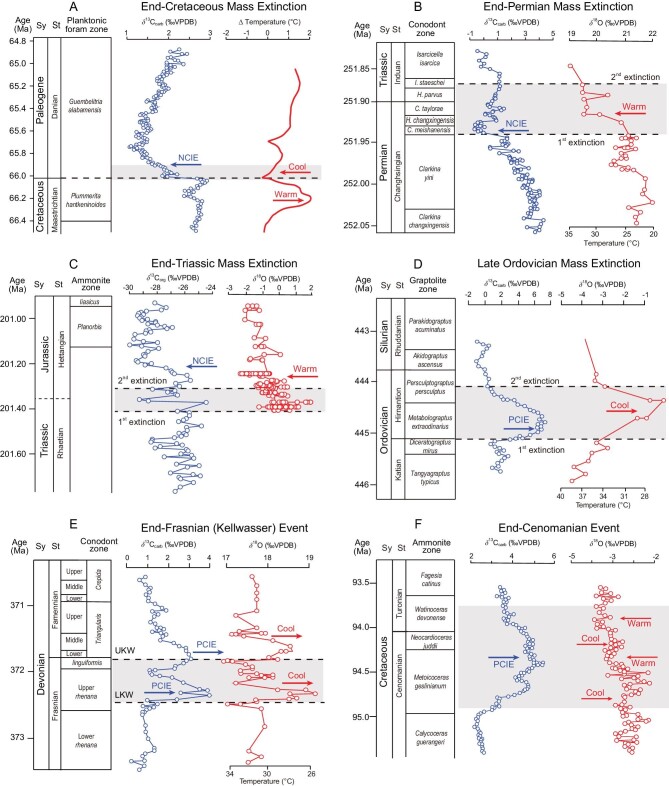
C-cycle (δ^13^C_carb_, δ^13^C_org_) and temperature (δ^18^O) proxy records for various mass extinctions. (A) End-Cretaceous Mass Extinction, or ECME (IODP Site U1403, Newfoundland [35]); (B) End-Permian Mass Extinction, or EPME (Meishan D, China, δ^13^C [36], δ^18^O [37]); (C) End-Triassic Mass Extinction, or ETME (St. Audrie's Bay, England, δ^13^C [38]; Lavernock Point, England, δ^18^O [39]); (D) Late Ordovician Mass Extinction, or LOME (Monitor Range, United States, δ^13^C [40], δ^18^O [41]); (E) End-Frasnian (Kellwasser) Event, part of the Late Devonian Mass Extinction, or LDME (Behringhauser Tunnel, Germany, δ^13^C [42], δ^18^O [43]); and (F) End-Cenomanian Event (Eastbourne, UK [44]). PCIE and NCIE represent positive- and negative carbon isotope excursions, respectively. *C*. = *Clarkina, H*. = *Hindeodus, I*. = *Isarcicella*. LKW = Lower Kellwasser; UKW = Upper Kellwasser; Sy = system; St = stage.

The LOME and LDME biocrises are fundamentally different from the ECME, EPME and ETME in ways that point to a completely different type of mechanism. In contrast to the complex short-term temperature changes of the ECME [35] (Fig. 2A) and the long-term (hyper)warming associated with LIP eruptions [45,46] (Fig. 2B–C), these mass extinctions were associated with long-term cooling trends that commenced tens of millions of years prior to the onset of mass mortality [47,48], and they both culminated in brief ice ages, i.e. the latest Ordovician Hirnantian [41] and end-Devonian Hangenberg glaciations [49]. Moreover, unlike the other three Big Five mass extinctions, these two biocrises were associated with large positive carbon-isotope excursions (PCIEs; Fig. 2D–E) indicating a fundamentally different response of the global carbon cycle (i.e. net carbon burial) from that of the ECME, EPME and ETME (whose NCIEs are indicative of net carbon release) (Fig. 2A–C). The LOME and LDME are also distinct in their ecological scope of operation (i.e. being limited mainly to tropical marine biotas) and their protracted, multistage character [3,50]. Finally, both of these biocrises had manifest temporal links to the evolution of land plants—specifically, the appearance and spread of bryophyte-grade plants during the Middle to Late Ordovician [51] and the rapid spread of vascular (especially seed) plants in the Late Devonian [52,53].

The principal alternative to an LIP mechanism as the trigger for the LOME and LDME is global climate change linked to the evolution of land plants, e.g. the ‘land-plant–weathering hypothesis’ [3]. This hypothesis links marine mass extinctions to the spread of land plants via their effects on continental weathering regimes. Specifically, as early plant clades spread across continents, they promoted chemical weathering via larger root systems, physical substrate disturbance via root penetration, and climate humidity via enhanced evapotranspiration. These weathering-related effects led to increased nutrient fluxes to marine environments, triggering massive algal blooms and water-column deoxygenation [3,54]. Evidence in support of this hypothesis has been accumulating for decades (Fig. 1), with studies documenting the evolution of early bryophyte-grade [51] and vascular plants [55], weathering rate intensification at the FFB [56,57], and increased terrestrial nutrient fluxes [58] and climate-floral relationships throughout the Devonian [59–61]. Significantly, the land-plant–weathering hypothesis [3,54] satisfactorily accounts for nearly all observational data related to the LOME and LDME events, which the bolide and LIP models do not. Although unambiguous evidence of the appearance of a key new plant taxon or clade immediately preceding a biocrisis such as the FFB is lacking, temporal relationships between paleobotanical developments and marine mass extinctions can be difficult to establish owing to limited documentation of the land-plant fossil record, causing punctuated migrations (e.g. [62]) to be overlooked. Thus, cause-and-effect sequences may be incorrectly inferred if a key plant taxon first appeared in a more poorly studied region and only later migrated into better-studied areas, an issue relevant to paleobotanical triggers but not to bolide and LIP mechanisms of mass extinction.

## TOWARDS A GENERAL THEORY OF MASS EXTINCTION CAUSATION

In many studies of mass extinctions, it is conventional to include a laundry list of possible causes and then assert that, in view of such a diverse set of possible mechanisms, the origin of the biocrisis of interest is not well understood and is, therefore, worthy of further study. More often than not, this approach is self-serving, disingenuous and problematic. It is self-serving in presenting a facile and weak rationale for a study. It is disingenuous in treating all published hypotheses as having equal merit when, for nearly all mass extinctions, existing research has narrowed the range of likely mechanisms and would permit more nuanced judgments concerning the relative merits of the remaining candidates. And it is problematic in that it almost invariably conflates proximate causation with ultimate causation, clouding the discussion of extinction mechanisms rather than providing clarity. This last issue, in particular, has long been a major impediment in developing a general theory of mass extinction causation.

‘Proximate causation’ refers to the immediate cause of death—it is akin to saying that a person died as a result of pulmonary congestion. ‘Ultimate causation’ refers to the initial cause (or ‘trigger’) in a chain of events leading to death—it is akin to saying that a person died as a result of Covid infection. With regard to extinctions, commonly cited proximate causes include a wide range of climato-environmental factors such as temperature change, oceanic anoxia and environmental acidification, either singly or in combination. These factors can trigger hyperthermia, suffocation, ionic imbalances and other adverse physiological effects in individual organisms, and, as individuals succumb, diminish the vitality and viability of populations of a species and of entire ecological communities. However, all such proximate causes are driven by some other, more remote (i.e. ultimate) cause. In this regard, it should be noted that similar collages of proximate climato-environmental changes can potentially develop from different ultimate causes, complicating determination of the latter. Unfortunately, many studies have cited proximate causes as the mechanism for a mass extinction seemingly without any awareness that such attributions provide little insight into the ultimate cause of a biocrisis. Yet the distinction between proximate and ultimate causation, which has been clearly articulated in relatively few studies (e.g. [63]), is absolutely critical to a systemic and holistic understanding of the phenomenon of mass extinction.

In the following, we will discuss proximate causation from the perspective of carbon-cycle controls, review evidence for the established types of ultimate causes (‘triggers’) of mass extinctions, examine mass extinction events with probable bioevolutionary triggers and tectono-oceanic triggers, and finally, consider other issues relevant to an understanding of mass extinction causation.

### Proximate causation

Proximate climato-environmental causes of biotic mortality are varied, with changes in temperature and ocean-redox conditions being among the most commonly cited in mass extinction studies. The importance of temperature is manifest in that all biotic species are adapted to a specific optimal temperature range [64], with shifts toward higher or lower temperatures having various, mostly harmful physiological effects [65,66]. Large temperature changes can trigger wholesale biotic migrations [67], with the migrations themselves contributing to mortality and extinction through disruption of ecological relationships [68]. Redox conditions are critical in all aqueous systems in that any environment characterized by intermittent or long-term de-oxygenation will experience the loss of most or all animal life [69]. Apart from temperature and redox changes, biotic stresses can result from various other proximate climato-environmental factors, including abrupt salinity changes (aqueous [70]), soil loss (terrestrial [71]), siltation (aqueous [72]) and environmental acidification (aqueous or terrestrial [73]), although these factors are rarely regarded as the dominant influence on biotic changes.

**Figure 3.**
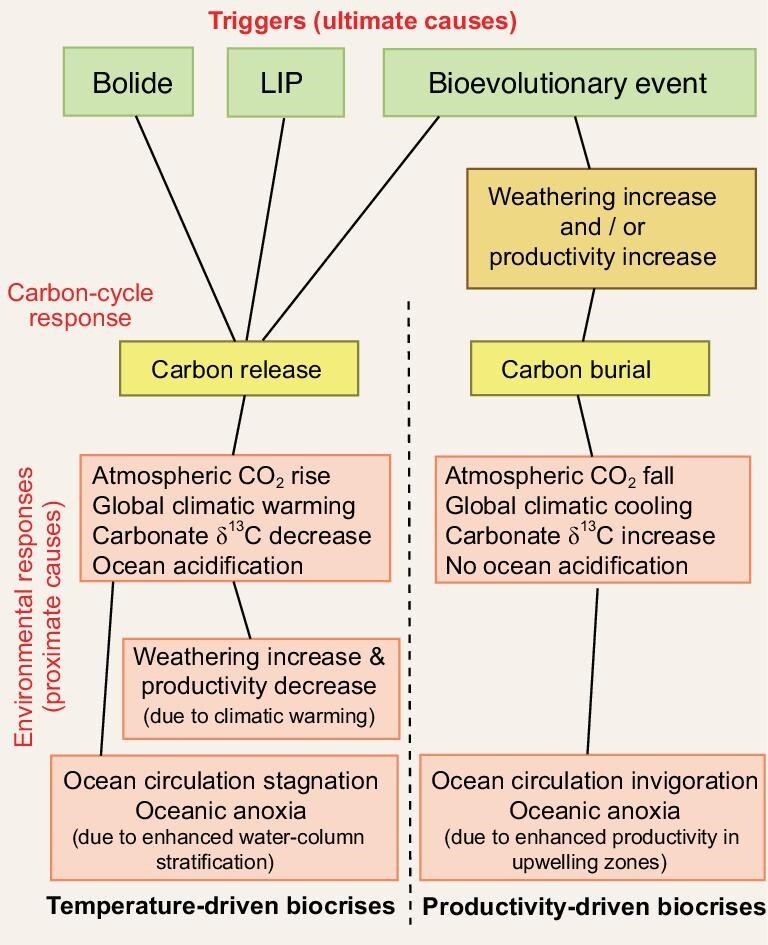
Generalized flowchart showing role of carbon-cycle response (yellow) in linking triggers (ultimate causes; green) to environmental responses (proximate causes; red) during major biocrises. For biocrises in which the trigger leads to carbon release (i.e. temperature-driven biocrises; left pathway), the resulting increase in atmospheric CO_2_ levels is responsible for changes in climate (i.e. warming), weathering intensity, marine productivity and other environmental effects. In contrast, for biocrises in which the trigger leads to carbon burial (i.e. productivity-driven biocrises; right pathway), carbon-cycle changes are dependent on an initial increase in terrestrial weathering and/or marine productivity (orange), and other environmental effects follow from the resulting decline in atmospheric CO_2_ levels and global cooling. Expansion of oceanic anoxia and intensification of continental weathering are commonly features of both models and therefore not diagnostic of either one.

Temperature and redox changes are generally related to perturbations of the global carbon cycle, making the latter a key player in most biocrises. The tangled web of proximate causation typical of many biocrises can be usefully dichotomized as related to either carbon release or carbon burial (Fig. 3). Specifically, climate change typically involves either cooling, which is associated with organic carbon burial and atmospheric CO_2_ drawdown, or warming, which is associated with organic carbon release and atmospheric CO_2_ buildup [74,75]. Indeed, these patterns are standard interpretations of changes in marine carbonate carbon isotope records, with PCIEs indicative of organic carbon burial and commonly linked to evidence of cooling and/or glaciation, and NCIEs indicative of organic carbon release and linked to evidence of hyperwarming [76].

Carbon-release events generally represent *‘temperature-driven biocrises’*, in which greenhouse gas buildup in the atmosphere causes climatic (hyper)warming and attendant environmental effects (Fig. 3). Release of carbon from the Earth's crust, rhizosphere and biosphere (in order of relative importance) can be triggered by bolide impacts (e.g. ECME; Fig. 2A), LIP eruptions (e.g. EPME or ETME; Fig. 2B–C) or biological activity (e.g. LQME). Although both bolide impacts and LIP eruptions may have short-term cooling effects linked to aerosol injection into the atmosphere (e.g. [77]), most proxy records at geological timescales show warming trends in their aftermath as a consequence of greenhouse gas release (Fig. 2A–C), which generally occurs over longer intervals and at a larger geographic scale than aerosol release [78]. The degree of warming associated with massive LIPs such as the Siberian Traps can be extreme (>10°C [37]) (Fig. 2B), with warming being the principal driver of longer-term (>1 Myr) environmental changes such as stagnation of oceanic circulation and intensification of the hydrological cycle, reduced marine productivity (reflected in NCIEs), increased continental erosion, and seawater acidification (the latter being linked to volcanic or evaporite sulfate emissions; e.g. [32]). This suite of features characterizes the ECME [79], EPME [80] and ETME [81] (Fig. 3).

In contrast, carbon-burial events are commonly *‘productivity-driven biocrises’*, in which increases in terrestrial and/or marine productivity result in enhanced organic carbon burial, leading to global cooling and attendant environmental effects (Fig. 2). The best-studied productivity-driven biocrises are the LOME, e.g. [41,82] (Fig. 2D) and the end-Frasnian event, e.g. [42,83] (Fig. 2E), both of which were associated with enhanced marine productivity and organic carbon burial (reflected in PCIEs [84,85]) as well as with productivity-driven expansion of oceanic anoxia [86,87] (Fig. 3). Both of these biocrises were preceded by long-term cooling trends [47,48] and culminated in abrupt short-term cooling episodes coincident with the main mass mortality events [41,49].

One environmental parameter that has proven ineffective in distinguishing temperature-driven from productivity-driven biocrises is oceanic redox conditions. In essence, most types of marine environmental perturbations promote ocean-redox shifts toward more reducing conditions (Fig. 3). Expanded anoxia during temperature-driven biocrises such as the EPME is the product of reduced overturning circulation and stronger water-column stratification [88] rather than increased marine productivity [89]. In contrast, expanded anoxia during productivity-driven biocrises such as the LOME, LDME and end-Smithian is largely a product of cooling-driven invigoration of global-ocean circulation, leading to intensified upwelling and productivity rates along continental margins [86,90,91]. Consequently, the distribution of oceanic anoxia tends to be weighted toward continent-margin upwelling zones during productivity-driven events (e.g. [91]), which differs from the pattern of whole-basin anoxia associated with temperature-driven events (e.g. [92]) (Fig. 3). Although short-term cause-and-effect relationships generally cannot be resolved in deep-time records, the association of oceanic anoxia with PCIEs and cooling is the signature of a productivity-driven biocrisis, in contrast to the NCIEs and climatic warming that characterize temperature-driven biocrises [76].

A second environmental parameter that has proven non-diagnostic of carbon-cycle perturbations is continental weathering. As with ocean-redox shifts, most types of terrestrial environmental perturbations lead to enhanced continental erosion and weathering fluxes. Increased weathering fluxes were associated with both temperature-driven biocrises such as the EPME [93,94] and productivity-driven biocrises such as the LOME [95,96], in the first case due to massive destruction of terrestrial vegetation and in the second to widespread glaciation (Fig. 3). On the other hand, the end-Frasnian crisis is associated with variable indicators of continental weathering changes, some studies documenting little change [56,97,98] and others strong change [99]. Several factors may be responsible for a lack of systematic relationships between weathering proxies and carbon-cycle perturbations during major biocrises: (i) local weathering intensity varies considerably at a broader scale [100]; and (ii) weathering intensity can vary inversely to globally integrated weathering fluxes due to landscape armoring effects [101].

### Ultimate causation

**Figure 4.**
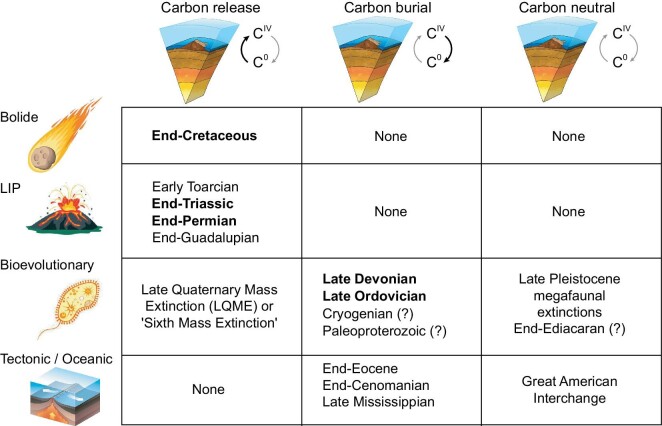
Classification of mass extinctions, based on a combination of ultimate (*y*-axis) and proximate (*x*-axis) causation. The Big Five Phanerozoic mass extinctions are shown in bold, and second-order biocrises in regular font. The occurrence of Cryogenian and Paleoproterozoic extinctions among microbial biotas remains speculative, and carbon-cycle changes during the end-Ediacaran were complex, as indicated by question marks. See text for discussion of specific extinction events.

The current status of research on mass extinction causation is that each of the Big Five biocrises has been robustly linked to one of three different ultimate mechanisms. In addition, there is some evidence to support a fourth mechanism, i.e. tectono-oceanic events such as the opening/closing of oceanic gateways, for certain second-order biocrises (see below). Based on the foregoing discussion, we propose that mass extinction causation is best considered from the joint perspective of both proximate and ultimate mechanisms. In this context, we propose a classification system of mass extinction events based on a combination of (i) the ultimate trigger and (ii) the pattern of carbon-cycle change (i.e. carbon release vs. carbon burial) (Fig. 4). In this scheme, biocrises known to have been triggered by a bolide impact or LIP eruption are exclusively carbon-release events owing to their fundamentally destructive natures, whereas biocrises triggered by bioevolutionary or tectono-oceanic processes can be variably carbon-release, carbon-burial or carbon-neutral events, depending on the specific relationships of each trigger to the global carbon cycle (Fig. 4). This classification accommodates all biocrises with well-established or tentatively known causations at present, although if the viability of another ultimate mechanism were demonstrated in the future, it could readily be accommodated through an expansion of this scheme.

Here, we consider four types of ultimate causes, or triggers, of mass extinctions: (i) bolide impacts, (ii) LIP eruptions, (iii) bioevolutionary events and (iv) tectono-oceanic events. In this survey, we offer relatively limited coverage of the bolide impact and LIP eruption mechanisms, which have been the subject of earlier in-depth reviews (e.g. [102] and [31], respectively). On the other hand, the roles of bioevolutionary and tectono-oceanic triggers in causing biocrises remain seriously understudied despite their probable importance through Earth history (especially the former), and thus they will be considered in greater detail. Although various extraterrestrial causes other than bolide impacts have been proposed (e.g. solar flares, gamma bursts and supernova explosions) and some of them have an extensive literature (e.g. supernovas; Fig. 1), there is little to no evidence that any of these mechanisms have actually caused a terrestrial biocrisis, and thus they will not be considered here.

#### Evidence for bolide impacts

There are ∼200 confirmed bolide impact craters on Earth (http://passc.net/EarthImpactDatabase/New%20website_05-2018/Index.html). However, craters are subject to destruction over time through erosion and subduction processes, and comparison with the impact records of the Moon, Mars and Mercury [103,104] makes clear that only a small fraction of terrestrial impact events have a preserved record. Bolide craters, and thus the impactors that generated them, vary widely in size, from small to immense [105], and only the latter can be expected to have had global biotic effects. Of all the known terrestrial impact events, the only one for which there is unequivocal evidence of a concurrent biocrisis is the ECME. One other possible candidate is the ∼34-Ma end-Eocene biocrisis, which resulted in modest extinctions among forams, mollusks and mammals [106–108]. The proximate cause of that event was unambiguously climate change (i.e. initial growth of the Antarctic icesheet and Southern Hemisphere cooling [109]), but whether the trigger was a bolide impact or merely a non-linear, threshold climate response to a long-term, tectonically driven decline of atmospheric CO_2_ (cf [110]) remains unclear. With regard to the former possibility, the 35.7-Ma Popigai crater [111] is probably too old to have played a role, but the 34.86-Ma Chesapeake Bay impact [112], which produced the widespread North American tektite strewn field (i.e. an impact ejecta layer [113]), may fit the bill. It is also possible that a combination of long-term CO_2_ fall and short-term impact-related cooling were responsible for triggering the end-Eocene biocrisis (see below).

Although an impact crater remains the *sine qua non* for a bolide strike, various types of sedimentological and geochemical features have proven highly useful in recognizing ancient meteorite strikes, a number of such events being known exclusively from diagnostic signatures in stratigraphic successions (e.g. [114–116]). Key sedimentological and geochemical features of bolide impacts include iridium anomalies, microspherule layers, quartz shock lamellae and fused breccia [12,117]. Of these proxies, iridium anomalies have proven somewhat problematic owing to microbial processes that concentrate that metal [118] and that may account for the majority of iridium anomalies in the rock record [e.g. 119]. Given the direct line that can be drawn between bolide impacts and these types of uniquely diagnostic mineralogic and geochemical features, it is unsurprising that the impact mechanism was the first to be thoroughly documented and widely accepted in the Earth sciences community.

#### Evidence for LIP eruptions

LIP eruptions have been numerous through Earth history, with >300 cataloged to date (http://www.largeigneousprovinces.org/record). As with impact craters, the known LIPs vary widely in size, from small to supermassive eruptions [120], among which only the largest can be expected to have had global biotic effects. Among the Big Five mass extinctions, both the EPME and ETME were unambiguously associated with large LIPS, i.e. the Siberian Traps [29] and the Central Atlantic Magmatic Province [30], respectively. However, more than a few second-order mass extinctions are suspected to have been caused by LIP eruptions, including the end-Guadalupian event (linked to the ∼259-Ma Emeishan LIP), the Early Toarcian event (linked to the ∼183-Ma Karoo-Ferrar LIP) and the end-Cenomanian event (linked to the ∼94-Ma Caribbean LIP) [121]. The end-Smithian biocrisis of the mid-Early Triassic, which was preceded by climatic hyperwarming [46], may also have had an LIP trigger (i.e. late-stage Siberian Traps eruptions [122]), although it is unclear whether the Late Smithian warming or the subsequent strong cooling across the Smithian–Spathian boundary was primarily responsible for this biocrisis [123]. In any case, the relationship between LIP eruptions and biocrises is firmly established, and an LIP origin must be considered a possibility for all mass extinctions.

In the case of LIP eruptions, Hg concentration anomalies and isotopes have recently been developed as an indicator of volcanogenic inputs to sedimentary successions, potentially providing a direct fingerprint [124–126]. As recently recognized, however, Hg anomalies can be produced by non-volcanogenic mechanisms, and caution must therefore be exercised in the interpretation of this proxy [127,128]. It should be noted that the case for LIPs as a mechanism of mass extinction was not based on the Hg proxy and, even today, does not depend primarily upon it. Rather, the case for LIPs was based largely on identification and dating of massive flood basalt deposits, demonstrating their coevality with certain biocrises [29,30].

Despite much research, an LIP mechanism for three of the Big Five mass extinctions (i.e. the ECME, LOME and LDME) remains speculative and contentious. Although the ECME event is known to have been approximately coeval with the Deccan Traps LIP, recent high-resolution dating has demonstrated that flood basalt eruptions in India commenced ∼250 kyr prior to the end-Cretaceous biocrisis [129–131], rendering cause-and-effect relationships uncertain. Imputation of an LIP trigger for the LOME and LDME is even more problematic. Despite much effort to identify contemporaneous LIPs, the evidence presented to date is underwhelming. Several LIPs of Late Devonian age have been reported, e.g. the Yakutsk–Viluyi, Kola–Dnieper and Pripyat–Dnieper–Donets LIPs [132], but the size of each is modest and all of them lack precise dating, casting doubt on their coevality with the end-Frasnian and end-Devonian biocrises. Furthermore, Hg anomalies in LOME and end-Frasnian sections offered as evidence of volcanic causation [132,133] have been interpreted uncritically and are likely not products of LIP-related Hg fluxes at all [128,134]. Most importantly, the LOME and LDME events show patterns of climato-environmental change that are incompatible with an LIP or bolide mechanism (see above).

#### Evidence for bioevolutionary triggers

Bioevolutionary triggers are likely to have played a much larger role in causing biocrises through time than generally realized. Nearly every major milestone in the history of the evolution of life, including both metabolic (e.g. oxygenic photosynthesis [135]; multicellular heterotrophy [136]) and biostructural innovations (e.g. adaptation of plants to life on land [137]), is likely to have led to a mass extinction as a result of attendant changes in environmental conditions and/or ecosystem organization. With regard to their carbon-cycle effects, bioevolutionary triggers may lead to net carbon burial, net carbon release or a carbon-neutral outcome, depending on the nature of the specific event (Fig. 3). Some bioevolutionary events resulted in a massive increase in carbon storage in the Earth's crust, e.g. the proliferation of oxygenic cyanobacteria during the Paleoproterozoic Great Oxidation Event (GOE), as recorded by the Lomagundi-Jatuli PCIE [138], whereas others have led to massive carbon release (e.g. the LQME) or no significant net change in carbon fluxes (e.g. the end-Ediacaran Event). The general underappreciation of the role of bioevolutionary triggers is due in no small part to the record of biocrises among microbial biotas in deep-time (i.e. pre-Phanerozoic) systems being poorly preserved and difficult to study.

The evidence for bioevolutionary triggers is typically even less direct than that for LIPs. Clues must be sought in extinction patterns (see below) and the identification of a plausible bioevolutionary trigger. A prime example of this approach is the Late Quaternary extinction of American megafauna, an event that exhibits biotic, geographic and temporal patterns that can be reasonably explained only as the result of the arrival of Paleolithic hunters in central North America around 15–13 ka [139]. Hypotheses for this biocrisis that invoke rapid climate change (or more exotic mechanisms like gamma bursts) fail to account for observations of spatial (limited to the Americas) and ecological selectivity (limited to megafauna), the lack of similar extinctions during multiple earlier Quaternary deglaciations, and the temporal relationship of this event to human migration into the Americas. With regard to other biocrises, a major environmental or ecosystem change can be a tip-off that a bioevolutionary trigger was responsible. For example, the sharp rise in atmospheric oxygen levels during the Paleoproterozoic GOE is highly likely to have precipitated a mass extinction among the obligate anaerobe microbial community that was dominant at that time [140]. In the case of the disappearance of the Ediacaran Biota around the Ediacaran–Cambrian boundary, the rapid appearance of hard parts among many unrelated marine invertebrate clades [141], a sudden increase in burrowing activity [142], and the appearance of bite marks/drillholes [143] are all clues pointing to the advent of the first active predators as an important agent of biotic turnover at that time.

### Biocrises with probable bioevolutionary triggers

#### Precambrian events

While the LOME and LDME are examples from among the Big Five mass extinctions of biocrises with a bioevolutionary cause [3,54], a number of other known or suspected biocrises had probable bioevolutionary triggers. Two of the more profound changes to the Earth-surface system through time were the large rises in atmospheric and oceanic oxygen levels accompanying the Paleoproterozoic GOE (∼2.4–2.0 Ga) and the Neoproterozoic Oxygenation Event (NOE; ∼850–540 Ma) [144]. Although evidence of mass extinction among the microbial communities of those eras remains scant [132], it is reasonable to infer that large rises in oxygen levels would have driven mass mortality owing to the detrimental effects of molecular oxygen on unprotected cells [145] and the toxicity of oxygen to the obligate-anaerobe prokaryotic communities of that era [146]. Moreover, these two events are known to have been associated with two of the major biotic transitions in Earth history, i.e. the appearance of eukaryotic organisms at ∼2.3 Ga [127] and that of metazoans at ∼0.7 Ga [147]. The evidence for contemporaneous massive carbon-cycle perturbations, a bellwether of major ecosystem changes, is manifest in the form of the Paleoproterozoic Lomagundi-Jatuli PCIE [138] and an unnamed >100-Myr-long Cryogenian interval of elevated δ^13^C_carb_ (>+5‰ [148]). Massive carbon burial during these events was accompanied by strong climatic cooling and glaciation, as recorded by the ∼2.4–2.2-Ga Huronian and ∼750–635-Ma Cryogenian ice ages [149,150], which are likely also to have stressed contemporaneous biotas. The associated patterns of climatic cooling and PCIEs demonstrate that the GOE and NOE were carbon-burial events (Fig. 3) and thus comparable to the LOME and LDME in their carbon-cycle effects (Fig. 4). Massive carbon burial during the GOE has been attributed to the spread of oxygenic cyanobacteria [135], which was a consequence of the shift from H_2_S to H_2_O as the dominant source of hydrogen in photoautotrophy [151], and during the NOE to either enhanced organic carbon burial by animals [152] or to enhanced marine productivity linked to increases in oxidative weathering and delivery of phosphorus to marine systems [153].

A biocrisis with uncertain climate implications is the demise of the Ediacaran Biota and the rise of the Cambrian Fauna around 540 Ma, at the end of the Ediacaran Period [154]. It has been broadly linked to global cooling [155,156] and expansion of marine anoxia [157], a pattern of environmental change that is suggestive of a carbon-burial event (Fig. 3), although it also coincided with the NCIE known as ‘BACE’ (BAsal Cambrian Excursion) [158] as well as sea-level rise [155], i.e. features that are more consistent with a carbon-release event. The conflicting C-isotope signals associated with the end-Ediacaran event may be because it was not primarily a carbon-driven biocrisis (Fig. 4). Indeed, the leading hypothesis to account for this biocrisis is the advent of active predators in marine ecosystems [141], an event that does not have an obvious connection to, and thus may have had a complicated relationship with, global carbon-cycle changes.

#### The Late Quaternary mass extinction (or ‘Sixth Mass Extinction’)

Most biologists and Earth scientists are in agreement that the Earth is on the threshold of another mass extinction, which has been termed the ‘Sixth Mass Extinction’ [159] but is herein named the LQME to conform to our terminology conventions. Choosing an appropriate temporal designation for this biocrisis is difficult because it spans the Late Pleistocene to Holocene and will extend into the soon-to-be-ratified Anthropocene Epoch [160], and given uncertainty about when the Quaternary Period will be over, ‘end-Quaternary’ seems inappropriate. Although not due to bioevolution per se, the LQME is the product of the technological evolution of hominids, i.e. the development of the advanced technologies that have given modern humans near-ubiquitous influence over the Earth's climatic, environmental and biospheric systems. In terms of its classification, the LQME is, to date, unique in representing a bioevolution-triggered carbon-release event (Fig. 4). Its specific suite of environmental effects is correspondingly unique, being characterized by greenhouse gas emissions, global warming and other features of temperature-driven crises, but also by massive erosion on land along with attendant effects on marine productivity and redox conditions that are more commonly associated with productivity-driven biocrises [161].

**Figure 5.**
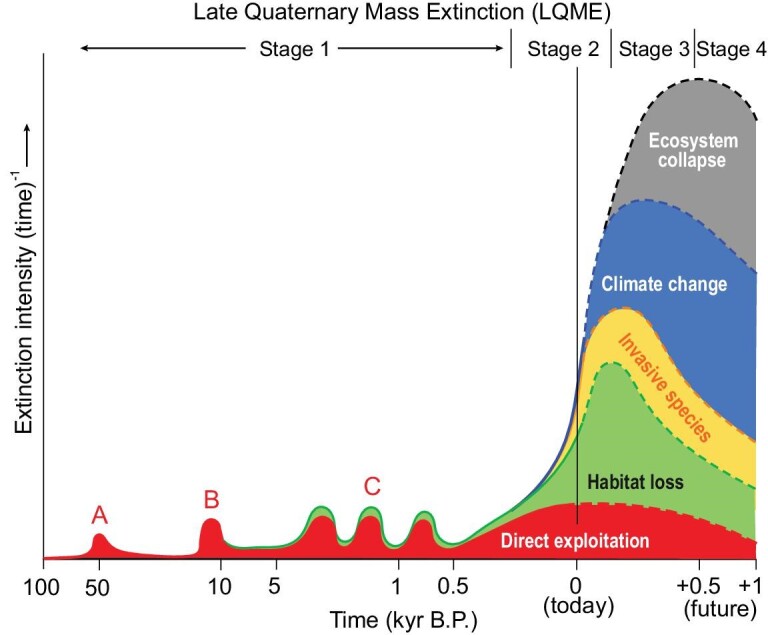
Stages of the Late Quaternary mass extinction (LQME). Stage 1 (from ∼50 to 0.25 ka), characterized by direct exploitation of species, comprised megafaunal extinctions in (A) Australasia, (B) the Americas and (C) the Indo-Pacific region. Stage 2 (from ∼0.25 ka to the near future) is dominated by extinctions due to habitat loss. Stages 3 and 4 (future; timeline speculative) will be marked by climate change and ecosystem collapse, respectively, as the dominant proximate causes of extinction, while invasive species will play a supporting role during Stages 2 to 4. In all stages, the technological evolution of humanity is the ultimate cause of biodiversity loss. Note that both axes have log scales, and that the y-scale is unquantified and relative. This figure is inspired by various literature sources cited in the text.

Owing to limitations on temporal resolution in stratigraphic successions and the inherent incompleteness of fossil records, the detailed history of most mass extinction events is poorly known. On the other hand, we have an outstanding record for the LQME, which is a complex, multiphase event that commenced at least 50 thousand years ago (Fig. 5). While the ultimate cause (i.e. human activity) has been invariant throughout this event, the main proximate cause of extinction has changed in the past and is likely to continue changing in the future. In its initial stage (roughly 50–0.25 ka), faunal extinction was due primarily to overexploitation of prey species, or to loss of top predators that were in competition with human hunters. It was marked by the disappearance of many members of regional megafaunas concurrent with the arrival of modern humans in previously unpopulated regions, e.g. Australasia at ∼50 ka [162], the Americas at ∼15–13 ka [163], Madagascar at ∼2 ka [164], New Zealand ∼800 years ago [165] and on various Indo-Pacific islands over the past few millennia [166]. In its present stage (0.25–0 ka), faunal extinction is due primarily to habitat loss, as humanity has transformed broad swathes of land surfaces to its own purposes, creating artificial ecosystems that are capable of sustaining only a fraction of the biodiversity of natural ecosystems [160], although other factors such as invasive species may be playing a key role in present-day species loss [167]. The first extinctions due to the direct effects of climate change (e.g. sea-level rise) have begun [168], and in the near future (i.e. over the next century or two; Fig. 5), climate change is likely to cull many species as they become increasingly maladapted to rapidly shifting climatic zones [169,170]. In its final stage, the LQME will probably be characterized by wholesale ecosystem collapse [171,172], as occurred during the largest biocrises of the past (e.g. [80]).

The intensity of faunal extinction will clearly vary through these stages—while the initial stage eliminated a significant fraction of large mammals in some regions, total biodiversity loss (as measured in species) was quite small at a global scale—only a fraction of 1% (Fig. 5). However, the ecosystem effects may have been significant, e.g. the elimination of large terrestrial herbivores, in particular, fundamentally altered some landscapes [173]. The present stage of the LQME has also driven a relatively small percentage of extant species to extinction, representing perhaps 1%–2% of total biodiversity (itself an uncertain quantity [174]), although with considerable variation across biotic clades, and with substantial uncertainty about the actual depth of the losses. These relatively modest biodiversity losses are belied by the pace of extinction, however, which is far above the natural background level (probably by a factor of 1000× or more [175]), representing a rate at which a large fraction of total biodiversity is likely to be lost over the course of the next few hundred years [176]. Furthermore, the most worrisome aspect of the present stage is that all ecosystems (marine, freshwater and terrestrial) have been seriously degraded, with large declines in population numbers and range contractions of many species (e.g. Living Planet Index; https://ourworldindata.org/grapher/global-living-planet-index), a pattern that presages much higher extinction rates in the not-too-distant future [177]. The final stage of the LQME—ecosystem collapse—will almost certainly be the grimmest reaper of all, with the potential to rapidly wipe out a large share of total global biodiversity [171,172] (Fig. 5).

### Biocrises with probable tectono-oceanic triggers

Tectono-oceanic processes, e.g. orogenic uplift, opening and closing of oceanic gateways, and latitudinal redistribution of continents and oceans, are capable of triggering large-scale climate changes in the Earth system, as shown by many events through Earth history (e.g. [178–180]). This mechanism is not considered to have been the ultimate cause of any of the Big Five mass extinctions, but it may have caused or contributed to several second-order biocrises (Fig. 4). Given that the underlying endogenic forces precipitating such changes operate at timescales of millions to tens or hundreds of millions of years at exceedingly slow rates, it might be expected that biotic communities adjust to the accompanying environmental changes through migration or gradual evolutionary processes. However, tectono-oceanic processes can trigger moderately rapid environmental changes by the crossing of a key tipping point or climatic threshold (e.g. one that triggers a rapid response, such as the onset of continental icesheet growth; cf [110]) or by the opening of continental pathways or oceanic gateways that result in faunal migrations or exchanges [181,182]. For all such events, a key question is whether such changes can proceed swiftly enough to create sufficient ecosystem stress to precipitate a biocrisis.

The Late Mississippian mass extinction, which occurred during the Serpukhovian Stage at ∼330–324 Ma, resulted in large losses of diversity among brachiopods [183] and reef fauna [184], although this biocrisis was at least partly due to low origination rates [185]. It coincided with a major expansion of continental ice mass in Gondwana [186], a large mid-Carboniferous eustatic fall that produced a deep unconformity in North America and elsewhere [187,188], and strong climatic cooling, as recorded in O-isotope records [184,189]. These features, accompanied by PCIEs [189], mark the Late Mississippian biocrisis as a carbon-burial event (Fig. 3). These observations suggest that global climatic cooling was the main cause of extinction. The trigger for this event remains uncertain, but the lack of any obvious candidate other than tectonism suggests that climatic cooling driven by uplift of the Appalachian-Hercynian orogens [190] reached a critical threshold around the end of the Mississippian (Fig. 4).

The ∼90-Ma end-Cenomanian mass extinction, which severely reduced diversity among rudistid bivalves, benthic foraminifera and ammonoids [191–193], was probably related to the opening of an oceanic gateway between the paleo-Southern Ocean and the Neotethys, in response to India's initial northward movement away from the other Gondwanan continents [194]. This event was associated with an unusual combination of global warming and higher marine carbonate δ^13^C values. Based on the former observation as well as widespread development of anoxia, an LIP trigger was proposed, although the source of magmatism has been variably cited as the Columbian-Caribbean Province, the High Arctic LIP, the Madagascar LIP or the Ontong-Java Plateau [195,196]. Although the Cenomanian–Turonian Boundary (CTB) interval was associated with peak oceanic spreading rates [197], the weakness of Hg enrichments throughout this interval [198] argues against an LIP trigger, and positive C-isotope anomalies (Fig. 2F) reveal it to have been a carbon-burial event rather than a carbon-release event (as is typical of LIPs). Changes in Nd-isotopic compositions around the CTB across the Atlantic to Tethyan regions provide evidence of major changes in global-oceanic circulation [199–201], consistent with the opening of an ocean gateway triggering this event (Fig. 4).

The ∼34-Ma end-Eocene biocrisis, which resulted in modest extinctions among forams, mollusks and mammals [106–108], was linked to rapid climatic cooling and initial growth of the Antarctic icesheet [109,202]. Although the proximate cause of the biocrisis was doubtlessly climate change, it is unclear whether the ultimate cause was a bolide impact or merely a non-linear, threshold climate response to a long-term, tectonically driven decline of atmospheric CO_2_ [110]. Several potential impactors have been identified, with the 34.86-Ma Chesapeake Bay impact [112] being a better fit temporally than the 35.7-Ma Popigai Crater [111]; the former event was large enough to produce the widespread North American tektite strewn field (i.e. an ejecta layer [113]). It is also possible that the end-Eocene biocrisis was a response to a combination of long-term CO_2_ fall and short-term impact-related cooling (see below).

A final example of what was certainly a tectonically driven biocrisis was the demise of various megafauna, especially marsupials in South America, during the ‘Great American Interchange’ [181], which was triggered by the formation of the Panamanian land bridge at 2.8 Ma [203]. This event led to nearly 25% of native South American mammalian genera going extinct, and by the end of the period of exchange more than half of the genera in South America were of North American origin (n.b., the effects of this event on the North American mammalian fauna were much smaller [181]). This biocrisis was clearly of tectonic origin and carbon-neutral in its carbon-cycle effects (Fig. 4).

### Other issues related to mass extinction causation

#### Discerning causation from biotic patterns

An undervalued approach to discerning proximate causation and its possible implications for ultimate extinction mechanisms is examination of the ecological patterns of mass extinction. Whereas extinction intensity tells us about the severity of a biocrisis, it provides no information concerning the types of ecological changes or the degree of ecosystem restructuring accompanying a mass extinction. The idea that biological selectivity and ecological patterns during an extinction event could be information-rich was proposed in the 1980s [204]. Subsequently, the hypothesis that morphological and ecological features promoting survival during mass extinctions differ from those favoring success during non-crisis intervals was formulated [205]. Ecological selectivity may be operating during the LQME [206], with extinction intensity predicted to be highest in high-latitude regions, but total species losses to be greater in the tropics due to the much higher biodiversity baseline [5].

Some general biotic patterns are evident in all biocrises. Taxon extinction probability increases with body size, due in part to larger animals being higher in the food chain and thus more susceptible to disruptions anywhere at lower trophic levels, and in part to smaller population sizes. One example of this is the disappearance during the EPME of all 25(+) species of gorgonopsids, the apex terrestrial predator of the Late Permian [207], whereas tetrapod clades at lower trophic levels (e.g. cynodonts, dicynodonts, procolonophonids and temnospondyls) survived in part [208]. Other known factors influencing survival probability include geographic range [209] and, for higher taxonomic levels, lower-order taxon richness (i.e. genera containing more species are less likely to go extinct [210,211]).

For the purpose of discerning ultimate causation, biotic patterns that are not shared by all mass extinctions are more useful than the general patterns discussed above. Based on an analysis of extinction selectivity during the EPME, it has been inferred that hypercapnia (high CO_2_) exerted a strong influence on marine invertebrate faunas, with carbonate-secreting organisms being especially susceptible as well as those with a high sensitivity of respiratory pigments and/or a limited ability to adapt metabolically to high or fluctuating CO_2_ levels [212,213]. Secondary environmental factors contributing to mortality included hyperwarming, anoxia and sulfide toxicity, with trophic stresses becoming acute at higher levels in the food web as ecosystem collapse proceeded [212–214]. An analysis of extinction patterns among fish during the EPME [215] concluded that broader tolerance to environmental variation was an important factor in survival, although factors such as a more active physiology and larger body size also conferred survival advantage. A key point is that the responses of individual taxa as well as entire ecosystems were driven by a confluence of environmental stresses that operated simultaneously, in some cases in mutually reinforcing ways [212,213].

#### Multiple causation of mass extinctions

Many studies have inferred multiple proximate causes for mass extinctions (e.g. [32]). Indeed, given that global catastrophes cause changes throughout the Earth-surface system, concurrent changes in temperature, ocean redox conditions, acidity and other environmental variables must be the norm. In most cases, it is difficult or impossible to tease apart the effects of multiple concurrent climato-environmental changes and determine their relative importance for an extinction event. What can be stated with assurance is that redox and temperature changes are regarded as having been particularly important in many Phanerozoic biocrises.

The issue of whether any mass extinctions had multiple *ultimate causes* is intriguing and as-yet unanswered. In general, invoking multiple causes for a single mass extinction appears to violate Occam's Razor, which favors the simplest possible explanation for a given set of observations. However, there are some examples in Earth history of biocrises with possible multiple causation—the most likely being cases where one factor has produced long-term environmental or ecosystem stresses that have amplified the effects of a second, unrelated factor. In this regard, the biocrisis that has received the most attention to date is the ECME, which coincided with both a bolide impact (the Chicxulub crater in Mexico) and an LIP (the Deccan Traps in India). Recent high-resolution dating has demonstrated that flood basalt activity in India spanned a ∼750-kyr interval bracketing the ECME [129–131], whereas the age of the Chicxulub crater exactly matches that of the biocrisis [216]. Thus, if the Deccan Traps eruptions played a role in the ECME, it was probably to generate widespread environmental stresses that made the effects of the Chicxulub impact more severe than they might otherwise have been [35], although the possibility of an eruptive stage exactly coinciding with the ECME [130] requires further investigation. The hypothesis that overlapping bolide–LIP events yield systematically higher extinction rates than either mechanism alone [217] is inherently plausible, although the number of such events is too small for a robust statistical evaluation.

The question of multiple causation has arisen also in the context of the LDME. The land-plant–weathering hypothesis [3] offered two scenarios: a ‘strong version’ in which both the long-term trends throughout the Devonian (i.e. climatic cooling, atmospheric CO_2_ drawdown) and the short-term biocrises of the end-Frasnian, end-Devonian and other horizons were due to land-plant bioevolutionary events, and a ‘weak version’ in which land-plant evolution merely created a backdrop of environmental stresses on which other events (e.g. bolide impacts or LIP eruptions) then operated episodically to produce extinction rate maxima. Although both possibilities remain under consideration, it should be noted that the end-Frasnian and end-Devonian biocrises were associated with strong short-term cooling and PCIEs [42,84], i.e. hallmarks of productivity-driven biocrises inconsistent with the temperature-driven processes that are typically associated with bolide impacts and LIP eruptions (see above). The potential productivity drivers of these short-term events are uncertain, although the Late Devonian coincided with the spread of archaeopterid forests and seed plants [3]. The latter development was particularly important because it permitted vascular plants to move out of moist lowland habitats and invade the drier upland regions that make up large parts of continental interiors, making it arguably the single most important evolutionary innovation in terms of areal expansion of land-plant habitat [53]. Thus, the ‘strong version’ of the land-plant–weathering hypothesis appears more likely at present, although this matter certainly warrants further investigation.

One objection to the land-plant–weathering hypothesis as a trigger of short-term biocrises relates to rates. Specifically, it has been argued that early land plants spread slowly across the globe over a time span of tens of millions of years and therefore could not have caused short-term biocrises such as the Kellwasser (end-Frasnian) and Hangenberg (end-Devonian) events, the estimated duration of which are between 50 kyr and a few hundred kyr [218,219]. This view reflects a misunderstanding of the nature of biotic expansions, which are generally not slow and continuous but episodic or ‘punctuated’ (note that the same debate regarding evolutionary tempo took place 50 years ago [220]). This point is illustrated by the history of humans: although their initial evolution in Africa was comparatively slow (>1 Myr), the subsequent spread of modern man (*Homo sapiens*) across the globe was much more rapid, i.e. into southwest Asia and southern Europe by 100 ka, into Australasia by ∼50 ka, into the Americas by ∼15–13 ka, across the Indo-Pacific at ∼3–1 ka, and into all remaining areas globally during the Age of Discovery (0.5–0 ka) [221] (Fig. 5). Although punctuated, the spread of modern humans has been rapid at geological timescales because of their high level of mobility—in the ancient world, jumps of plant and animal species across oceans may have occurred more sporadically in time. It also illustrates a general pattern that, although the evolution of a new taxon or clade may be protracted on its landmass of origin, subsequent expansions in range (i.e. across newly colonized landmasses) are rapid events. This latter point is well-illustrated by the rates of spread of modern invasive species across newly invaded continents, a process that typically requires 10^1^–10^3^ years [222], i.e. a timescale that is effectively instantaneous from a geological perspective (Fig. 5).

#### Periodicity in the mass extinction record

One area of mass extinction research that is closely linked to the bolide impact model is the issue of periodicity (or ‘quasi-periodicity’, as argued in some studies). The hypothesis of a ∼26-Myr periodicity in Phanerozoic biocrises was first promulgated by Raup and Sepkoski [204,223], with variations on this theme invoking other periodic intervals (e.g. [224]). A key assumption of any periodic mechanism is that all extinctions must have had the same ultimate cause. Most such hypotheses have invoked astronomical controls, e.g. the orbit of an unidentified companion star (‘Nemesis’) through the Oort Cloud, vertical motions of the Solar System across the galactic plane, or rotation of the Milky Way Galaxy (see review in [225]), although it also has been suggested—rather improbably—that non-astronomical intrinsic biological mechanisms could yield extinction periodicity [226]. However, quite apart from the statistical problems of such analyses (which are addressed in [227]), the fact that Phanerozoic mass extinctions have been triggered by multiple, fundamentally different and unrelated mechanisms effectively precludes any form of periodicity in their distribution through time. Although ‘mass extinction periodicity’ is still a popular topic in the literature (e.g. [228], Fig. 1), it is well past time to discard the idea that there is any temporal regularity to the extinction record.

#### Long-term changes in mass extinction causation

This survey of mass extinction causation reveals a feature of potential interest, i.e. that over the course of Earth history there has been a transition from dominantly carbon-burial to dominantly carbon-release events (Fig. 4). This is indisputably the case for the Big Five mass extinctions, among which those associated with carbon burial (i.e. LOME and LDME) preceded those associated with carbon release (i.e. EPME, ETME and ECME). Even though some exceptions to this pattern can be found among the second-order biocrises, it is nonetheless noteworthy. This pattern is perhaps to be understood in the context of the ‘Earth as fuel cell’ theory of [229], in which the Earth's biosystems have reduced carbon and effected its net transfer from the atmosphere-hydrosphere to the lithosphere over several billion years, charging the planetary fuel cell, after which the crustal reservoir of reduced carbon has been repeatedly tapped by various processes that have released a portion of the stored energy. These carbon-tapping processes include bolide impacts, LIP eruptions, and now anthropogenic use of fossil fuels. In this regard, the shift from dominantly carbon-burial biocrises to carbon-release biocrises toward the end of the Paleozoic Era represents the development of a highly charged planetary fuel cell, a condition that may have been associated with ‘peak organic carbon burial’.

## FINAL THOUGHTS

Scientific progress has benefitted enormously from application of the principle of multiple working hypotheses [230]. However, the literature on mass extinction causation is strewn with poorly tested hypotheses that have been uncritically adopted for many biocrises. Some of the hypothesized triggers for mass extinction events have lived on well past their sell-by dates, despite the fact that overwhelming evidence points in other directions. One example of this is the Late Quaternary extinction of megafauna in the Americas. As noted in [139], ‘the overkill hypothesis, at least in general terms, already has been ‘proven’ as thoroughly as any historical hypothesis can be. All of the key evidence was available years ago, and all of it firmly refutes competing, ecologically oriented hypotheses. The event's timing, rapidity, selectivity and geographic pattern all make good sense according to the anthropogenic model, and no sense at all otherwise.’ It is a form of false equivalence that all extinction mechanisms must be given equal weight in a scientific study, much as we see in the media today regarding political issues, that all sides must be given equal time in the limelight. More nuanced judgments are needed, and failed ideas should be jettisoned. In this regard, the ‘one size fits all’ approach to the analysis of mass extinction causation should be permanently laid to rest. It is long since time to acknowledge that extinctions in Earth history have multiple ultimate causes. Apropos of this, the perspicacious words of the American author F. Scott Fitzgerald are called to mind: ‘The test of a first-rate intelligence is the ability to hold two opposing ideas in mind at the same time and still retain the ability to function’ [231]. In the present instance, the intellectual task is less onerous—what is needed is not the ability to reconcile two opposing ideas but, rather, to recognize that two or more causation mechanisms for mass extinctions can simultaneously be valid.
